# Proteomic and Metabolomic Analyses Reveal Contrasting Anti-Inflammatory Effects of an Extract of Mucor Racemosus Secondary Metabolites Compared to Dexamethasone

**DOI:** 10.1371/journal.pone.0140367

**Published:** 2015-10-23

**Authors:** Samuel M. Meier, Besnik Muqaku, Ronald Ullmann, Andrea Bileck, Dominique Kreutz, Johanna C. Mader, Siegfried Knasmüller, Christopher Gerner

**Affiliations:** 1 Department of Analytical Chemistry, University of Vienna, Währinger Straße 38, 1090, Vienna, Austria; 2 Syntrion GmbH, Robert-Bosch-Straße 6, 75365, Calw, Germany; 3 Institute of Cancer Research, Medical University of Vienna, Borschkegasse 8a, 1090, Vienna, Austria; Chang-Gung University, TAIWAN

## Abstract

Classical drug assays are often confined to single molecules and targeting single pathways. However, it is also desirable to investigate the effects of complex mixtures on complex systems such as living cells including the natural multitude of signalling pathways. Evidence based on herbal medicine has motivated us to investigate potential beneficial health effects of *Mucor racemosus* (M rac) extracts. Secondary metabolites of M rac were collected using a good-manufacturing process (GMP) approved production line and a validated manufacturing process, in order to obtain a stable product termed SyCircue (National Drug Code USA: 10424–102). Toxicological studies confirmed that this product does not contain mycotoxins and is non-genotoxic. Potential effects on inflammatory processes were investigated by treating stimulated cells with M rac extracts and the effects were compared to the standard anti-inflammatory drug dexamethasone on the levels of the proteome and metabolome. Using 2D-PAGE, slight anti-inflammatory effects were observed in primary white blood mononuclear cells, which were more pronounced in primary human umbilical vein endothelial cells (HUVECs). Proteome profiling based on nLC-MS/MS analysis of tryptic digests revealed inhibitory effects of M rac extracts on pro-inflammatory cytoplasmic mediators and secreted cytokines and chemokines in these endothelial cells. This finding was confirmed using targeted proteomics, here treatment of stimulated cells with M rac extracts down-regulated the secretion of IL-6, IL-8, CXCL5 and GROA significantly. Finally, the modulating effects of M rac on HUVECs were also confirmed on the level of the metabolome. Several metabolites displayed significant concentration changes upon treatment of inflammatory activated HUVECs with the M rac extract, including spermine and lysophosphatidylcholine acyl C18:0 and sphingomyelin C26:1, while the bulk of measured metabolites remained unaffected. Interestingly, the effects of M rac treatment on lipids were orthogonal to the effect of dexamethasone underlining differences in the overall mode of action.

## Introduction

The search for optimized drugs for the treatment of specific diseases has created large and successful industries. Typically, a key mechanism responsible for the occurrence of a disease state is targeted by identifying interaction partners and pharmaceutically active molecules which might interfere in these signalling pathways [1]. It is certainly true that almost all acute disease symptoms can currently be controlled using specific drugs, some of which had been established upon application of such strategies. On the other hand, it is evident that chronic diseases also represent a serious burden to the public health which needs to be managed with strategies other than classical medications [2]. Actually, drugs used in chronic treatment schemes like antihistamines may lose effectiveness upon repeated or high-dose usage. The use of complex mixtures from natural products may thus represent a reasonable alternative to cope with the development of tolerance to the drug. However, the scientific verification of effectiveness of such remedies still represents an experimental challenge due to the large number of analytes that constitute the formulation and due to the requirement of holistic experimental approaches required to elucidate and characterize beneficial pharmacological effects. This is one of the reasons why cell model systems grew popular in the early phases of drug discovery and development [3].

It is well acknowledged that microorganisms produce a large number of still largely unknown secondary metabolites with intriguing properties for pharmaceutical activity [4]. In order to make a better use of this resource, the large scale synthesis of such compounds has been suggested [5]. In most cases however, experience based on rather poorly documented folk medicine represents the initiation point for research activities, *e*.*g*. herbs used in traditional Chinese and European medicine. Not only herbs have been applied in folk medicine, but also products derived from microorganisms are used. Indeed, current studies suggest anti-inflammatory effects of compounds derived from *candida albicans* [6] and other microorganisms [7]. In particular, *Mucor racemosus* (M rac) is a common mould which is known to produce metabolites with potential pharmacological effects [8,9]. The potential effectiveness of this extract for the alleviation of inflammatory processes is investigated in this study.

Due to their involvement in chronic diseases, the investigation of inflammatory processes is in the focus of our proteomics research activities. We have characterized inflammation-induced proteome alterations in primary human peripheral blood mononuclear cells using a 2D-PAGE based top down-approach [10] as well as by applying a mass spectrometry based bottom up-approach [11]. Quite recently, we have demonstrated that targeted proteomics is a valid strategy for the quantification of cytokines and chemokines using human umbilical vein endothelial cells (HUVECs) as model system [12]. Analysis of pharmacological effects on cells *via* proteome profiling and targeted proteomics offers a unique opportunity for the investigation of complex samples such as natural extracts because alterations of the cellular proteome reflect changes in the molecular response of cells as living entities. Furthermore, the proteomics assays were complemented with a validated metabolomics assay, which is also based on LC-MS analysis [13]. Here we applied and comprehensively evaluated these MS-based proteomics and metabolomics approaches for the investigation of the inflammatory modulating properties of the complex extract derived from M rac.

## Materials and Methods

### Extract of M rac

Cell supernatant extracts of *Mucor racemosus* (M rac) were provided by Syntrion GmbH (German GMP reference No.: 25–5483.0-1/Syntrion, National Drug Code USA: 10424). M rac extracts have market authorization and are sold as SyCircue. The extracts were obtained using a GMP-approved pharmaceutical production line and a standardized manufacturing process that has been validated according to EU guidelines for the production of pharmaceuticals. In brief, the supernatants were extracted with pharmaceutical grade saline solution. Culture media-free total cell preparations are incubated with saline solution and subsequently filtered twice through 0.2 μm pharmaceutical grade filters to obtain an extract of secondary metabolites free from microorganisms.

### PBMCs and Cell Lines

Peripheral blood mononuclear cells (PBMCs) were isolated from venous blood as described in reference [11] with written consent of each donor and approval of the Ethics Committee of the Medical University of Vienna (Application 2011/297 by C.G. entitled:”Characterization of inflammatory activated mononuclear cells of the peripheral blood and the effects of dexamethasone and salicylic acid on the protein expression. An exploratory study on 20 volunteers”. Second, primary human umbilical vein cells (HUVEC) were purchased from Lonza (Walkersville Inc., USA). Cells were cultured in endothelial basal medium supplemented with the EGM-2 SingleQuot Kit (both EBM, Lonza Walkersville Inc., USA), 10% FCS and 100 U/mL penicillin/streptomycin (both ATCC, USA) according to the instructions of the manufacturer. Experiments were performed up to passage six for HUVEC, respectively, in 6-well plates. Viability was routinely assessed by a standard Trypan blue exclusion assay and was consistently better than 98%. The biological samples were prepared in triplicates to allow statistical analysis.

### Stimulation and Treatment

PBMCs were inflammatory stimulated with 1 μg/mL lipopolysaccharide (LPS) and 5 μg/mL phytohaemagglutinin (PHA) for 2D-PAGE analysis and cultured for six hours in medium containing ^35^S-methionine and ^35^S-cysteine for metabolic labeling of newly synthesized proteins [10]. The stimulated cells were further treated after 1 h with the M rac extract at a dilution of 1 : 10’000 for 6 h. 2D-PAGEs of control, control + M rac, inflammatory stimulated and inflammatory stimulated + M rac were performed.

HUVECs were inflammatory stimulated with 10 ng/mL interleukin 1-beta (IL-1β, Sigma-Aldrich, USA) for 24 h. For 2D-PAGE analysis, the cells were cultured in medium containing ^35^S-methionine and ^35^S-cysteine for metabolic labeling of newly synthesized proteins for six hours (18–24 h). The cells were stimulated for 1 h and then treated with a dilution of 1 : 10’000 of M rac for further 23 h. Again, 2D-PAGEs of control, control + M rac, inflammatory stimulated and inflammatory stimulated + M rac were performed. For MS-based shotgun proteomics, cytoplasmic fraction and supernatants of the control, stimulated and/or treated HUVECs were isolated and the proteins were digested by standard trypsin digestion. For obtaining supernatants, cells were washed once with PBS and cultured in 1.5 mL serum-free medium for 6 hours after treatment. Subsequently, the supernatants were filtered through 0.2 μm filters (Whatman, Germany) and stored at –80°C until sample preparation. For targeted proteomics, the cells were additionally treated with dexamethasone (100 ng/mL) and the recently published method was followed to quantify cytokines and chemokines in supernatants. For targeted metabolomics analysis, cell lysates of prepared HUVECs were obtained using 85% ethanol in 10 mM phosphate buffer following Application Note 2001–1 (Biocrates Life Sciences AG, Innsbruck, Austria).

### Tryptic Digestion

HUVEC cytoplasmic proteins were pre-fractionated in-gel by SDS-PAGE and subsequently digested using trypsin following standard protocols [11]. Proteins of supernatants were in-solution digested with Trypsin/LysC according to reference [12]. The dried samples were stored at –20°C before LC-MS/MS measurement.

### 2D-Gel Electrophoresis

Cytoplasmic HUVEC and PBMC samples were loaded by passive rehydration on IPG strips pH 5–8, 17 cm (Bio-Rad, Hercules, CA) at room temperature. Isoelectric focusing (IEF) was performed in a stepwise fashion (1 h 0–500 V linear; 5 h 500 V; 5 h 500–3500V linear; 12 h 3500 V). After IEF, the strips were equilibrated with 100 mM DTT and 2.5% iodacetamide according to the instructions of the manufacturer (Bio-Rad). For SDS-PAGE, the Protean II xi electrophoresis system (Bio-Rad) was used. IPG strips were placed on top of 1.5 mm 12% polyacrylamide slab gels and overlaid with 0.5% low-melting agarose with bromophenol blue. After electrophoresis, gels containing ^35^S-labeled proteins were stained with a 400 nM solution of Ruthenium II tris (bathophenanthroline disulfonate) as described before [14]. Fluorography scanning was performed with the FluorImager 595 at a resolution of 100 μm. After scanning the fluorescence, the gels were dried for subsequent autoradiography in order to differentiate newly synthesized proteins. Dried gels were inserted into cassettes including a phosphor screen as detector for β-radiation of the ^35^S-labeled proteins. These phosphor screens were scanned with the PhosphorImager SI MAC (Molecular Dynamics) with 100 μm, see also reference [15]. Proteins were identified by LC-MS/MS analysis of proteolytic digests of isolated spots from the 2D-PAGE as described previously [10].

### Shotgun Proteomics

Trypsin-digested HUVEC cytoplasmic fractions and supernatants were analysed with an XCT-Ultra ion trap mass spectrometer equipped with a chip cube for nano-flow LC (Agilent) as described previously in detail [16]. The peptides were separated using a 60 min gradient from 0–40% ACN and analysed via peptide fragmentation. The MS/MS data was searched against the SwissProt/UniprotKB protein database (version 10/2012).

For the determination of sample complexity, 1 μL of a 1 : 10 dilution of M rac was injected into an Agilent 1290 UHPLC Infinity system using a 4.3 cm x 2.1 mm reversed phase column (Phenomenex) with 1.8 μm particle size. A gradient was formed with 0.1% formic acid in water (phase A) and 0.1% formic acid in 80% ACN (phase B) from 0–60% B during 15 min. A Q Exactive orbitrap was used for mass spectrometric analysis in positive ionisation mode.

### Targeted Proteomics

Targeted proteomics was performed according to our recently published method [12]. Prior to LC-MS/MS analysis, four synthetic standard peptides were added to each sample (10 fmol/μL each) including Glu1-Fribrinopeptide B (Peptide Sequence: EGVNDNEEGFFSAR), M28 (Peptide Sequence: TTPAVLDSDGSYFLYSK), HK0 (Peptide Sequence: VLETKSLYVR) and HK1 (Peptide Sequence: VLETK(ε-AC)SLYVR). The trypsin digested supernatants were analyzed by a nano-Chip-LC using a 1260 Infinity Series HPLC system (Agilent) coupled to a 6490 triple quadrupole mass spectrometer (Agilent). A large capacity protein chip (G4240-62010) with a 160 nL enrichment column and a 150 mm x 75 μm separation column (5 μm ZORBAX 300SB-C18, 30 Å pore size) was used and 1 μL of the sample was injected. Mobile phase A consisted of 97.8% H_2_O, 2% ACN and 0.2% FA, mobile phase B of 99.8% acetonitrile and 0.2% formic acid. A flow rate of 5 μL/min was applied for sample loading *via* the capillary pump and 400 nL/min for peptide separation *via* the nano pump. A 20 min gradient was applied for peptide separation while the total run time was 40 min including column regeneration. Total peak areas of peptides were exported from Skyline (Version 2.5, Ref. [17]) as csv-files and normalization was performed in Excel.

### Targeted Metabolomics

Targeted metabolomics was performed using the AbsoluteIDQ p180 kit (Biocrates Life Sciences AG, Innsbruck, Austria). The kit allows the identification and (semi) quantitation of 188 metabolites including 40 acylcarnitines, 42 amino acids and biogenic amines, the sum of hexoses, 15 sphingolipids and 90 glycerophospholipids by LC- and flow injection analysis (FIA)-MRM on an AB SCIEX Qtrap 4000 mass spectrometer and an Agilent 1200 RR HPLC system, which were operated with Analyst 1.6.2 (AB SCIEX). The chromatographic column was provided by Biocrates. The samples including blanks, seven calibration standards and quality controls were prepared according to the manufacturer. Quality controls were analyzed after every 20^th^ sample. All amino acids and biogenic amines were derivatized with phenylisothiocyanate. The experiments were validated with the supplied software (MetIDQ, Version 5-4-8-DB100-Boron-2607, Biocrates Life Sciences, Innsbruck, Austria) and data analysis was performed with the in-built data evaluation features (StatPack). The correlation coefficients of the calibration curves of the amino acids and biogenic amines were between 0.9604 (glutamic acid) and 0.9997 (threonine). Internal standards of putrescine, SDMA and DOPA were not observed during the LC-MRM analysis and these analytes were rejected. Additionally, the quality controls revealed that the accuracy of the internal standards of asparagine, glycine and histamine were outside the limits and were rejected as well. Finally, 166 metabolites were successfully validated and considered for data evaluation.

Cell lysates of control, inflammatory activated (IL-1β) and inflammatory activated plus treated (dexamethasone or M rac) HUVECs were analysed in two biological and two technical replicas.

## Results

Extracts of M rac were obtained from cell supernatants using a GMP-approved production line and are used as sold. First, the mutagenic and genotoxic potential of the M rac extract was assayed prior to proteomic investigations. Ames tests on five *Salmonella typhimurium* strains, TA 98, 100, 102, 1535 and 1537, showed that the extract is devoid of mutagenic activity by showing colony numbers that were at the frequency of spontaneous revertants up to 500 μL/plate. The extract was diluted 1 : 10 for the Ames tests and was then added in 50, 100, 175 and 500 μL/plate. Additionally, the spontaneous revertants were always in the expected range (S1 File, see also S1, S2 and S3 Tables). Metabolic activation using S9 fractions did not increase the frequency of spontaneous revertants. Moreover, the extract of M rac did not display any antiproliferative activity against human peripheral lymphocytes and human derived hepatoma cells (HepG2) at doses between 0.1–10 μL/mL and the total viability was always >98%. Again, the extract was diluted 1 : 10 prior to the analysis. HepG2 cells possess active phase I and phase II metabolic enzymes, while peripheral lymphocytes do not. Addition of the S9 fraction to the peripheral lymphocytes did not increase the antiproliferative activity of the extract. Therefore, the M rac extract does not display mutagenic or genotoxic potential, nor acute toxicity at a dilution of 1 : 10.

**Fig 1:**
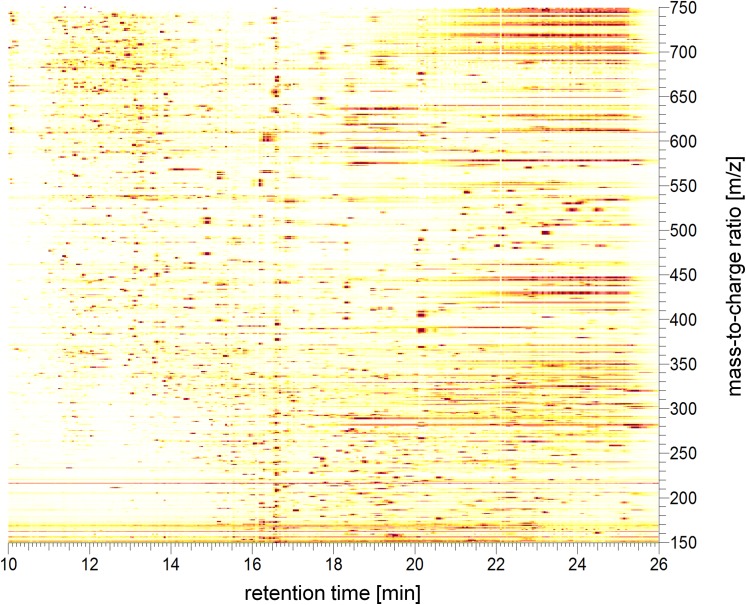
Two-dimensional representation of an LC-MS analysis of the *M rac* extract. The analytes were separated using HPLC and detected with a Q Exactive orbitrap in the positive ion mode. Each spot annotates a distinct molecular feature, which can be counted dependent on the applied sensitivity threshold. Several hundred different constituents can easily be distinguished. The x-axis shows the retention time between 10 and 26 min and the y-axis shows the mass range of *m/z* 150–750.

LC-MS/MS analysis of the M rac extract itself provided some indication with respect to the complexity of the mixture (Fig 1). The dilution used here was three orders of magnitude higher than the applied concentration for the cell treatment. Over a range of 16 min, several hundred molecular features were detected, each of which could potentially play a role in the therapeutic action of the extract. It was not yet attempted to characterize and quantify the bioactive molecules in the M rac extract. At this stage, interpretation of the high resolution LC-MS experiment could even be misleading because of the different ionization efficiencies of molecules. Furthermore, each bioactive molecule requires a certain concentration for exerting a biological or pharmacological effect. This implies that the most abundant features in the LC-MS run are not necessarily the ones responsible for the observed effects in the subsequent experiments. It is exactly the purpose of this study, to investigate the potential beneficial effects of the M rac extract as a whole.

**Fig 2:**
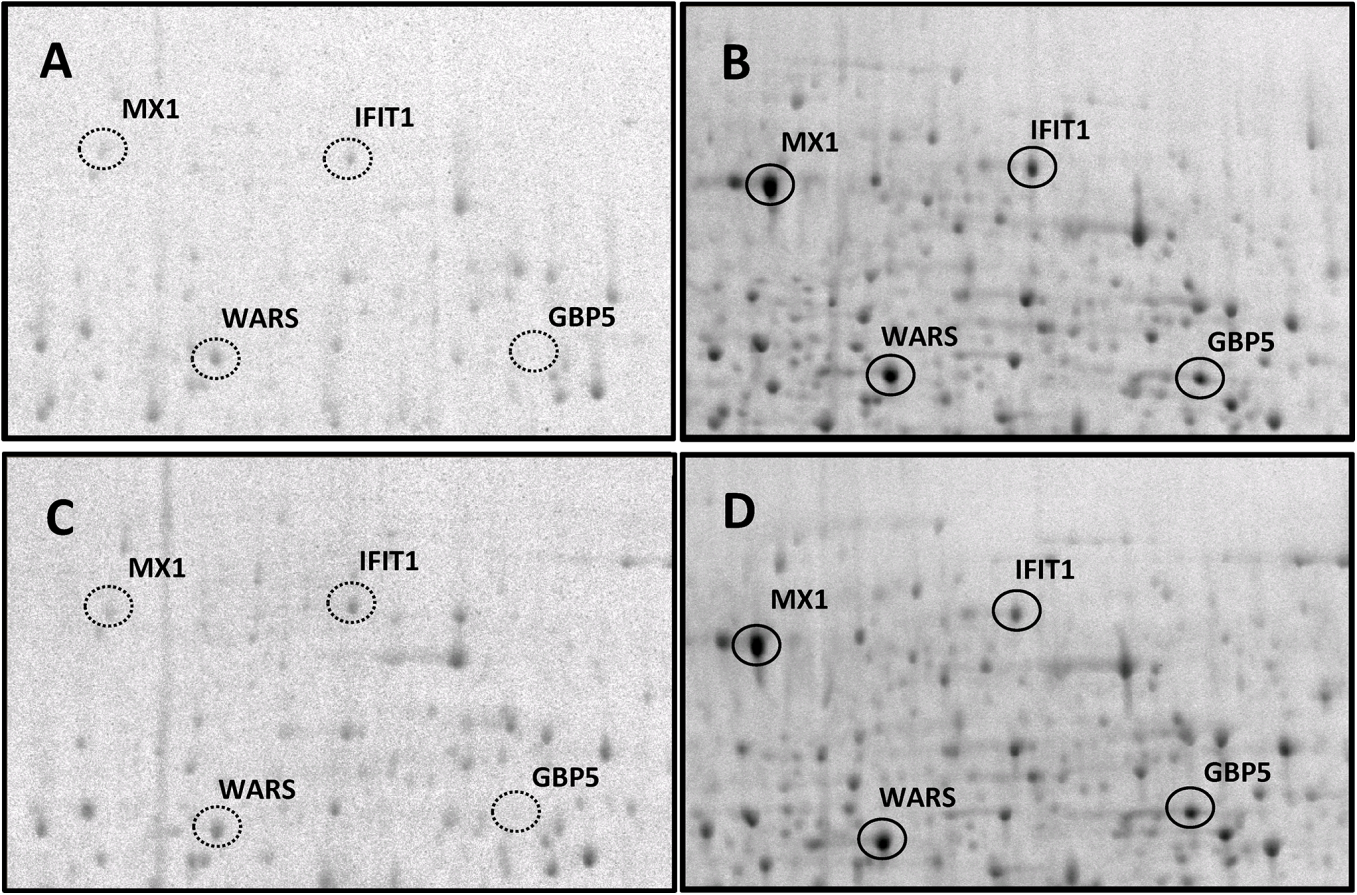
2D-PAGE of cytoplasmic proteins isolated from peripheral blood mononuclear cells (PBMCs). Cells were metabolically labelled while being inflammatory stimulated and treated with *M rac* extract (1:10’000 dilution), proteins were detected by autoradiography. A) untreated control. B) inflammatory stimulation with LPS and PHA strongly induced inflammatory mediators such as MX1, IFIT1, WARS and GBP5. C) treatment of PBMCs with *M rac* extract hardly induced any proteome alterations. D) treatment of stimulated PBMCs with *M rac* extract attenuated the induction of IFIT1, while other inflammation markers remained largely unchanged. Proteins were identified by proteolytic digestion of each spot and subsequent LC-MS/MS analysis.

The modulation of inflammatory processes by M rac extracts was first tested on inflammatory stimulated peripheral blood mononuclear cells (PBMCs). Freshly isolated PBMCs were stimulated with lipopolysaccharide (LPS) and phytohaemagglutinin (PHA) and cultured in medium containing ^35^S-methionine and ^35^S-cysteine for metabolic labelling of newly synthesized proteins as described previously [10]. Thereafter, the stimulated cells were treated with the M rac extract at a dilution of 1 : 10’000. Cytoplasmic proteins from unstimulated and stimulated PBMCs, both untreated and treated with the diluted M rac extract, were separated by 2D-PAGE and subjected to autoradiographic detection, which provides a measure for protein synthesis in the cytoplasm of the cells [18]. Autoradiographic intensities of many known pro-inflammatory mediators displayed strong up-regulation upon inflammatory stimulation as expected (Fig 2A and 2B), including MX1, IFIT-1, GBP5 and WARS [11]. Treating unstimulated cells with M rac extracts had no effect on protein synthesis (Fig 2C), while treatment of the stimulated cells resulted in the apparent down-regulation of inflammation-associated proteins such as IFIT-1 (Fig 2D).

**Fig 3:**
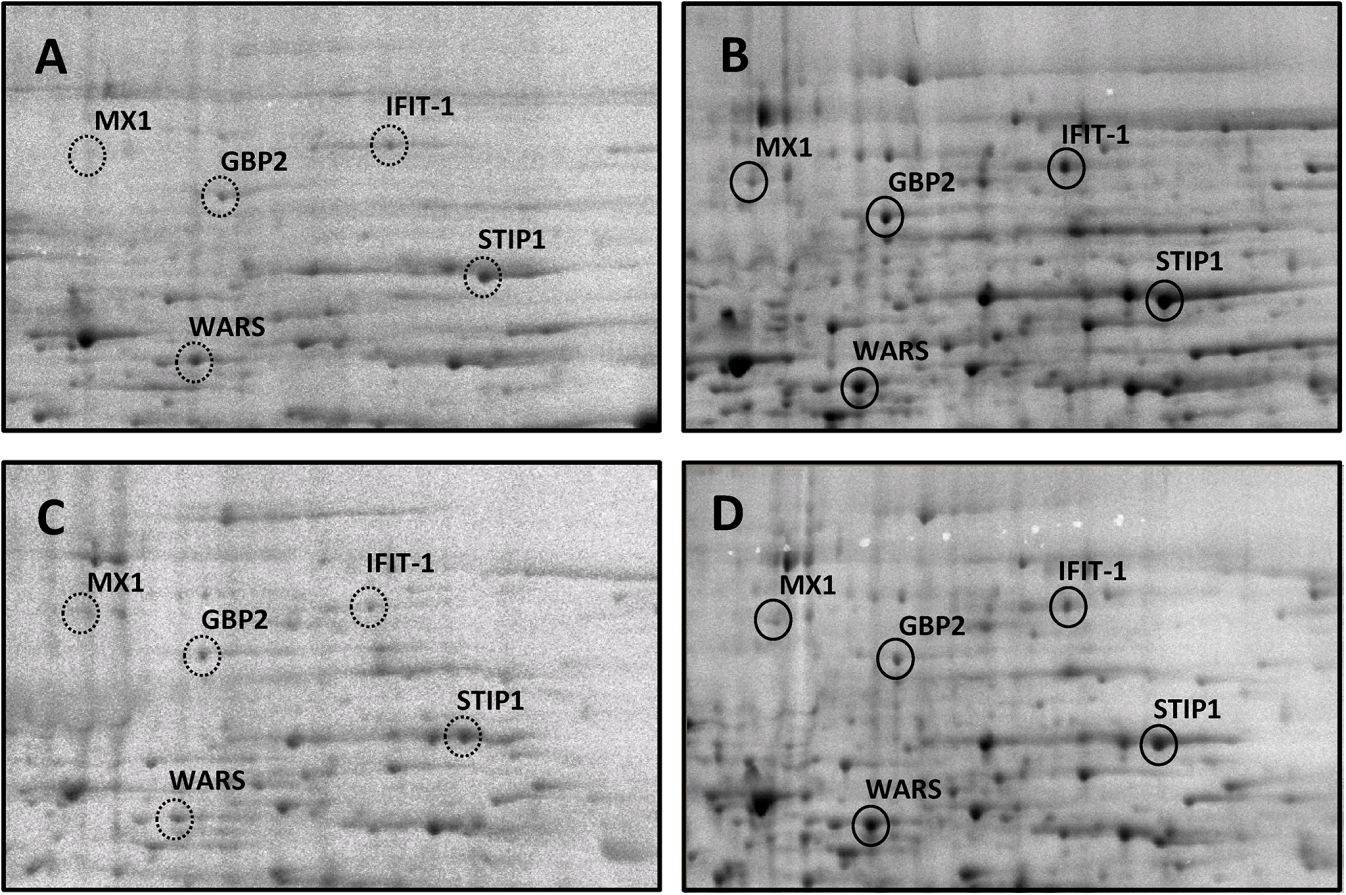
2D-PAGE of cytoplasmic proteins isolated from human umbilical vein endothelial cells (HUVECs). Cells were metabolically labelled while being inflammatory stimulated and treated with *M rac* extract (1:10’000 dilution), proteins were detected by autoradiography. A) untreated control. B) inflammatory stimulation with interleukin 1-beta strongly induced inflammatory mediators such as MX1, IFIT1, WARS and GBP2 as well as the stress-related protein STIP1. C) treatment of HUVECs with *M rac* had little effect on the synthesis of cytoplasmic proteins. D) treatment of stimulated HUVECs with *M rac* extract attenuated the induction of IFIT1, GBP2 and STIP1. Proteins were identified by proteolytic digestion of each spot and subsequent LC-MS/MS analysis.

As these results were encouraging, we proceeded to test primary human endothelial cells (HUVECs), which are important players in chronic inflammation, in an analogous experimental setup [19]. In this case, inflammatory stimulation was accomplished by treating the cells with interleukin 1-beta (IL-1β). Again treatment of unstimulated cells with M rac extracts had little effect on the synthesis of cytoplasmic proteins in 2D-PAGE compared to HUVEC controls (Fig 3A and 3C). Moreover, inflammatory stimulation resulted in a strong up-regulation of protein synthesis of similar proteins as in PBMCs, *e*.*g*. the inflammatory mediators MX1, IFIT-1 and WARS, but also GBP2 and the stress-related protein STIP1 (Fig 3B). Remarkably, the stimulated endothelial cells showed a more pronounced responsiveness to M rac extracts, and all five mentioned inflammatory mediators were down-regulated upon treatment (Fig 3D). These findings indicate that stimulated endothelial cells might be a particular target for treatment with the M rac extract SyCircue.

In order to confirm the inflammatory modulating effects of the M rac extract, we applied MS-based label-free shotgun proteomics on cytoplasmic and secreted proteins isolated from HUVECs. Untreated HUVECs were investigated, as well as inflammatory stimulated and stimulated with M rac treatment. The extract was applied at the same dilution of 1:10’000 and similarly for all subsequent LC-MS experiments. In total, 1382 proteins were identified over all experiments with two or more peptides. Corroborating our results obtained from 2D-PAGE, all proteins indicated in the gels were also identified using the shotgun approach. Furthermore, the emPAI values (exponentially modified protein abundance index [20]) of the cytoplasmic proteins GPB2, MX1, STIP1 and IFIT1 were increased in the inflammatory stimulated cells in comparison to the control cells and then down-regulated upon treatment with M rac (Table 1).

**Table 1:** Selected identification data obtained from shotgun analysis of cytoplasmic and secretome fractions of HUVECs. Accession, Uniprot Accession number; Gene, gene name, Peptide IDs, number of distinct peptide identification; C, cytoplasmic fraction; S, secretome fraction. A semi-quantitative abundance measure is provided via emPAI values. Con, untreated cells; IL1b, stimulated with Interleukin 1-beta; m rac, stimulated with Interleukin 1-beta and treated with *M rac* extract. While inflammatory stimulation increased almost all emPAI values of the listed inflammatory mediators, treatment with *M rac* extract of stimulated cells resulted in the down-regulation of most emPAI values.

Accession	Protein name	Gene	Peptide IDs	emPAI con	emPAI IL1b	emPAI m rac
P20591	Interferon-induced GTP-binding protein Mx1	MX1	C: 6	0.0	0.348	0.136
P09914	Interferon-induced protein with tetratricopeptide repeats 1 (IFIT-1)	IFIT1	C: 2	0.064	0.064	0.0
P32456	Interferon-induced guanylate-binding protein 2	GBP2	C: 1	0	0.054	0.0
P31948	Stress-induced-phosphoprotein 1 (STI1)	STIP1	C: 9	0.147	0.136	0.044
P05121	Plasminogen activator inhibitor 1 (PAI-1)	SERPINE1	S: 35	5.271	6.342	2.775
P26022	Pentraxin-related protein PTX3	PTX3	S: 16	1.395	3.007	0.995
P05231	Interleukin-6 (IL-6)	IL6	S: 8	0.0	1.72	0.848
P10145	Interleukin-8 (IL-8; C-X-C motif chemokine 8)	CXCL8	S: 4	0.0	2.728	2.728
P19875	C-X-C motif chemokine 2	CXCL2	S: 5	0.0	2.69	0.778
P42830	C-X-C motif chemokine 5	CXCL5	S: 3	0.0	2.205	0.0
P09341	Growth-regulated alpha protein	CXCL1	S: 6	0.0	2.594	1.783
P09603	Macrophage colony-stimulating factor 1 (CSF-1)	CSF1	S: 2	0.0	0.179	0.132

Remarkably, several pro-inflammatory cytokines and chemokines, including IL-6 and CXCL1, 2, 5 and 8 were specifically identified in the secretome upon inflammatory stimulation with IL-1β, which were not found in the control cells. Treatment of inflammatory stimulated HUVECs with the M rac extract then caused a significant down-regulation of IL-6, CXCL1, 2 and 5, but not IL-8. Additional pro-inflammatory mediators were also affected, namely SERPINE1, PTX3 and CSF-1. The regulation of these secreted inflammation mediators is also exemplified by the emPAI values in Table 1.

**Fig 4:**
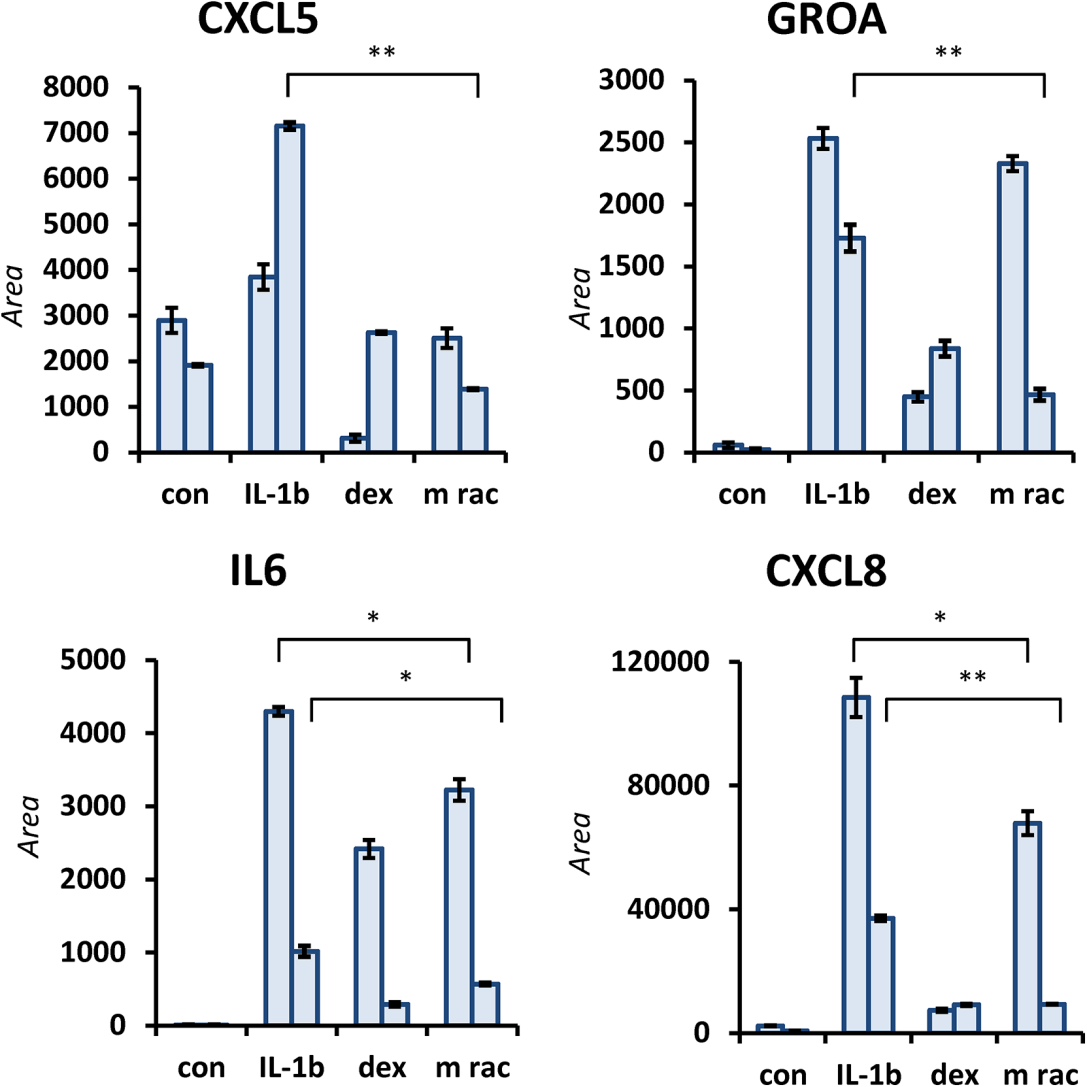
Targeted proteomic analysis of pro-inflammatory cytokines secreted by inflammatory stimulated and treated HUVECs. Experiments were performed using two biological with two technical replicates. Inflammatory stimulation (IL-1β) up-regulated the levels of all cytokines in comparison to untreated controls (con). Treatment with dexamethasone (dex) down-regulated all cytokines as expected for a strong anti-inflammatory drug. Remarkably, also treatment with *M rac* extract was capable of significantly down-regulating these cytokines (M rac compared to IL-1β: *, p<0.01; **, p<0.001). “Area” refers to the area under the curve of the chromatographic peak in the nLC-MRM experiment of the quantifier transition of each peptide.

The findings obtained from label-free shotgun proteomics were further supported by targeted proteomics using relative quantitation of protein regulations by MRM mass spectrometry. These nLC-MRM experiments give information on the retention and fragmentation of specific proteolytically obtained peptides. The resulting area under the curve of each peptide can then be compared in different conditions. Inflammatory activation of HUVECs resulted in an increase of secreted cytokines and chemokines, *e*.*g*. CXCL5, GROA, IL-6 and IL-8. Treatment with the M rac extract also significantly down-regulated the secretion of the inflammatory cytokines IL-6, CXCL5 and GROA (Fig 4, S4 and S5 Tables). Interestingly, targeted analysis also revealed IL-8 to be significantly down-regulated upon treatment with M rac extracts, in contrast to the results from shotgun proteomics. The targeted proteomics approach was also used to compare the inflammation modulating effects of the M rac extract to the one of dexamethasone, which is a routinely applied anti-inflammatory agent. For this purpose, inflammatory stimulated HUVEC cells were treated dexamethasone at a concentration of 100 ng/mL and cell supernatants were isolated, proteolytically digested and analyzed by nLC-MRM analogously. Indeed, dexamethasone treatment successfully down-regulated CXCL5, GROA, IL-6 and IL-8 in the endothelial cells, conform with its known way of action in PBMCs [11,12]. Both the M rac extract and dexamethasone produced a very similar down-regulation of pro-inflammatory cytokines and chemokines, however, dexamethasone did this to a greater extent than M rac.

**Fig 5:**
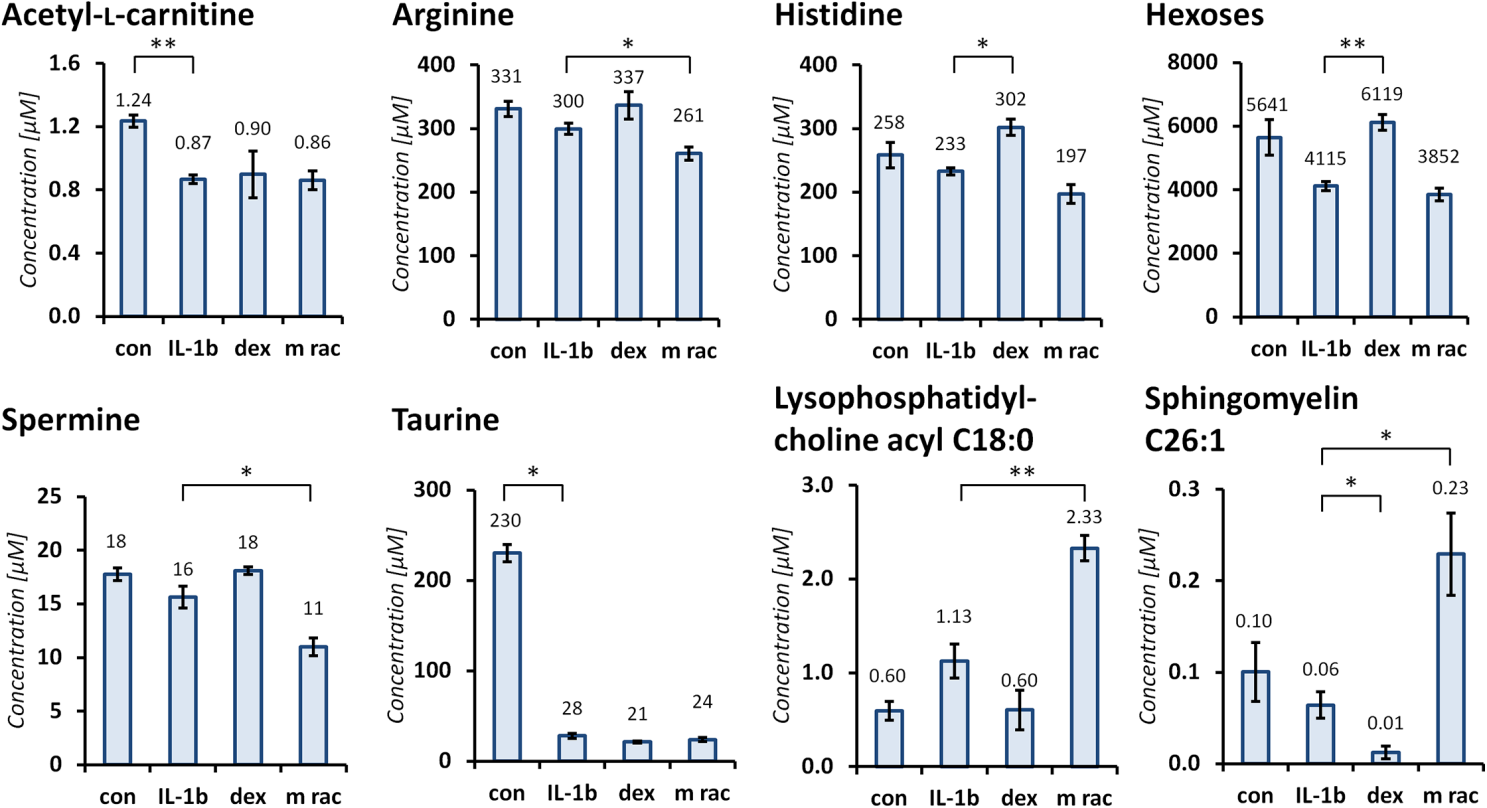
Regulation of selected metabolites assessed by the AbsoluteIDQ p180 metabolomics kit illustrating the contrasting inflammation modulating effects of dexamethasone (dex) and M rac. Again, HUVECs were stimulated with IL-1βeta (IL-1β) and additionally treated with dexamethasone or M rac extract (M rac). Error bars are derived from two technical and two biological replicates. (p-values: *, p<0.05 and **, p<0.005.)

Finally, we investigated inflammation-modulating effects in HUVEC cells by a targeted metabolomics approach. For this purpose, we compared cell lysates of untreated controls, of inflammatory activated cells and inflammatory stimulated cells, which were treated with the M rac extract and dexamethasone, respectively. After protein precipitation, the soluble fraction containing the metabolites was derivatized with phenylisothiocyanate. The p180 kit returned 166 validated metabolites for data evaluation. Most measured acyl-l-carnitines, amino acids and biogenic amines were not regulated upon inflammatory stimulation or subsequent treatment. Exceptions are acetyl-l-carnitine, arginine, histidine, spermine, and taurine, as well as the sum of hexoses. Treatment with the M rac extract had significant effects on metabolite concentration changes of arginine (p-value = 0.028) and spermine (p-value = 0.013) compared to inflammatory stimulation, and both metabolites were further down-regulated, while dexamethasone treatment re-established initial metabolite concentrations (Fig 5). More detailed information on regulation profiles and significances can be found in S6 Table. Acetyl-l-carnitine and taurine were both significantly down-regulated upon inflammatory stimulation, but did not change by treatment neither with dexamethasone nor the M rac extract. The kit additionally allowed the assessment acyl lysophosphatidylcholines (lysoPCs), diacyl phosphatidylcholines (PC aa’s), acyl alkyl phosphatidylcholines (PC ae’s) and (hydoxy)sphingomyelins (SMs). Inflammatory stimulation of HUVECs resulted in an up-regulation of numerous diacyl and acyl alkyl phosphatidylcholines across the whole series, but not lysoPCs or sphingomyelins significantly. Subsequent treatment of inflammatory activated HUVECs with dexamethasone then down-regulated 84% of the phosphatidylcholines between 2.2 and up to 5-fold. This effect was even more pronounced in the class of sphingomyelins, which were down-regulated 2–8-fold upon treatment with dexamethasone, while lysoPCs were not regulated. In contrast, treating stimulated HUVECs with the M rac extract led to a slight up-regulation of diacyl and alkyl acyl phosphatidylcholines but only a few in a statistical significant manner. Interestingly, also some (lyso)phosphatidylcholines and sphingomyelins were up-regulated with respect to inflammatory stimulated HUVECs. The most up-regulated lipids of activated HUVECs and subsequent M rac treatment were lysophosphatidylcholine acyl C18:0 (2.1-fold, p-value = 0.0023), hydroxysphingomyelin C22:1 (3.0-fold, p-value = 0.014) and sphingomyelin C26:1 (3.6-fold, p-value = 0.030).

## Discussion

Inflammation is a biological process employing many cell types and a great variety of molecules including proteins [11], metabolites [21] and lipids [22]. Especially chronic inflammation is a great challenge for the modern health system, and treatment options are desirable [23]. When evaluating complex mixtures with respect to potential anti-inflammatory effects, molecular profiling methodology proves to be a very suitable approach for gaining mechanistic insights into a cell’s responsiveness because standard biochemical assays are usually focussed on single analytes and are not able to capture the effects of a multitude of potentially bioactive molecules at once. To study a mixture such as the M rac extract containing several hundred secondary metabolites (Fig 1) requires a stable production over batches, which is assured by a GMP-approved production line. The extract was tested for mutagenic or potential toxic effects in Ames tests and in classical cell viability assays in order to exclude deleterious effects on the investigated cells during any further studies. The extract was devoid of any mutagenic or toxic properties at a dilution of 1 : 10 and volumes up to 500 μL/plate, which at least thousand-fold exceeded the concentrations used during proteomics and metabolomics studies. Therefore, we conclude that the contents of the extract did not exert toxic effects during the follow-up experiments and that the observed effects are pharmacologically relevant.

During the initial 2D-PAGE experiments, we observed a particular responsiveness of inflammatory stimulated HUVECs to M rac treatment, which was stronger compared to PBMCs. Of the cytoplasmic proteins from HUVEC cells, we observed a down-regulation of inflammatory mediators such as IFIT-1, WARS and MX-1, while only IFIT-1 was apparently down-regulated in PBMCs upon treatment. MS-based shotgun analysis of the proteome is a powerful technique in order to obtain a comprehensive overview on proteome alterations upon treatment of cells with stimulants. The results of these investigations corroborated the previous observations and allowed us to select the most biologically relevant marker molecules of the cytoplasmic fraction. Indeed identical proteins were down-regulated as in the 2D-PAGE experiment and their regulations are exemplified by the emPAI values in Table 1. Additionally, secretome profiling revealed cytokines and chemokines such as IL-6 and CXCL1 as important paracrine mediators of inflammation in accordance with an earlier study [11] and were down-regulated upon M rac extract treatment of inflammatory stimulated endothelial cells. However, due to the intrinsic nature of peptide identification in the data dependent acquisition mode, a relatively high coefficient of variation is observed. In order to gain statistical significance, we employed a targeted proteomics strategy which has been recently developed to measure the secreted cytokines and chemokines with high accuracy [12]. Here, we selected IL-6, IL-8, GROA and CXCL5 as meaningful marker molecules for following the modulation of inflammation.

M rac treatment induced significant attenuation of the four inflammatory mediators IL-6, IL-8, GROA and CXCL5 in inflammatory stimulated endothelial cells as revealed by targeted proteomics. These cells are part of the stroma, long-lived and capable of stimulating effector cells such as T-cells or macrophages [24] and as such, they are main players in chronic inflammation. Therefore, M rac extracts seem to possess the capacity of modulating chronic inflammation. Intriguingly, the anti-inflammatory properties of the M rac extract were more pronounced in the endothelial cells compared to PBMCs. As the latter are involved in acute inflammation, it seems that the M rac extract possesses also cell-type selectivity for chronic inflammation.

Dexamethasone is recognized as one of the most potent anti-inflammatory drugs widely applied in clinical practise and for this reason it was used as a positive control for the targeted analysis of the secretome of HUVECs. Both, dexamethasone and the M rac extract down-regulated the four previously mentioned inflammatory mediators in a significant fashion (Fig 4). While dexamethasone at a concentration of 100 ng/mL was more effective in the down-regulation of the cytokines and chemokines than the applied M rac extract at a dilution of 1 : 10’000, both treatments clearly showed the same trends. This is in clear contrast to results obtained from targeted metabolomics. Here, dexamethasone and M rac treatment showed partly orthogonal effects.

Inflammatory activation of HUVECs and subsequent treatment with M rac affected a smaller number of metabolites compared to dexamethasone and a selection is represented in Fig 5 (and S6 Table). On the one hand, the acyl carnitines, amino acids and biogenic amines remained relatively constant upon inflammatory stimulation and subsequent treatment with dexamethasone or the M rac extract. However, several metabolites of these classes were found to be regulated: Spermine is a negative mediator of inflammation as it can inhibit the synthesis of pro-inflammatory cytokines, *e*.*g*. IL-6 [25]. Inflammatory activation slightly decreased spermine concentration, while treatment with dexamethasone re-established metabolite levels identical to the control. On the contrary, treatment with the M rac extract even further down-regulated spermine concentrations as compared to the inflammatory activation. At first sight, this seems contradictory to the anti-inflammatory properties of the M rac extract. However, it is improbable that a single metabolite may be responsible for inflammation regulation. This may point to a different mode of action of M rac treatment, which culminates in the down-regulation of pro-inflammatory cytokines and chemokines. Acetyl-l-carnitine and taurine seem to be indicators of inflammatory activation as they are significantly down-regulated upon stimulation with IL-1β, however, subsequent treatment with either dexamethasone as a positive control or the M rac extract did not regulate these metabolite concentrations any further. Moreover, the lipids showed a more distinct regulation pattern across lipid classes. Inflammatory activation of the endothelial cells up-regulated specific members of diacyl and acyl alkyl phosphatidylcholines over the entire series, while lysophosphatidylcholines and sphingomyelins were not affected. Treatment with dexamethasone globally down-regulated the diacyl and acyl alkyl phosphatidylcholines, while treatment with the M rac extract slightly up-regulated these metabolites, albeit only a small number in a statistically significant fashion. The sphingomyelins were not significantly regulated upon inflammatory stimulation. However, a drastic down-regulation of 13 sphingomyelins (of a total of 16) up to 8.6-fold was observed after treatment with dexamethasone, while treatment with M rac led to a slight overall up-regulation (S6 Table). Specifically, hydroxysphingomyelin C22:1 and sphingomyelin C26:1 were significantly up-regulated 3- and 3.6-fold, respectively, by M rac treatment. Interestingly, M rac treatment led to slight and in some cases significant up-regulation of several lysophosphatidylcholines, while dexamethasone did not. For example, lysophosphatidylcholine acyl C18:0 (LysoPC C18:0) was shown to induce the production of IL6 or IL8 in smooth muscle cells [26]. Inflammatory activation increased LysoPC C18:0 concentrations and dexamethasone treatment re-established initial metabolite levels. Treatment with the M rac extract significantly increased LysoPC C18:0 even further.

Little is known about the effects of dexamethasone on the metabolome of inflammatory stimulated cells. Up-to-date, studies rather focussed on the influence metabolite administration on inflammation than metabolite regulation upon drug treatment. This leads to the above-mentioned apparent contradictive findings related to spermine and also LysoPC C18:0 [25,26]. However, it was found in an *in vivo* model that down-regulation of phosphatidylcholines may be due to increased formation of triglycerides upon dexamethasone administration [27]. In the same study, protein contents and phospholipids apparently decreased per gram tissue, which is in accordance to the down-regulations of phosphatidylcholines by dexamethasone in HUVEC cell cultures in this study. The metabolome of cell lysates represents intracellular activities rather than the paracrine effects exerted by cytokines and chemokines. We observed that dexamethasone treatment almost completely abrogated inflammatory effects on both, proteome and metabolome levels in HUVECs by down-regulating the secretion of cytokines and chemokines as well as by down-regulating diacyl and acyl alkyl phosphatidylcholines and sphingomyelins. M rac treatment down-regulated paracrine activities similarly to dexamethasone, *i*.*e*. the secreted cytokines and chemokines, but seemed to further enhance the intracellular effects of inflammatory activation at the level of metabolites. While both treatment options can be expected to have similar anti-inflammatory consequences, this finding points to rather different mode of action. This in turn may contribute to different effects on long-term applications, which may merit further investigations as long-term application of dexamethasone may lead to immune suppression [28]. However, further systems biology approaches will be required to elucidate the differences in the modes of action of dexamethasone and M rac treatment on inflammatory stimulated cells in more detail.

## Conclusions

We demonstrate that M rac extracts of secondary metabolites are powerful regulators of inflammatory processes. These effects were most pronounced in primary human endothelial cells, thus offering potential treatment options for chronic inflammation. Remarkably, metabolic consequences of inflammatory stimulation were found even more pronounced after M rac treatment, while dexamethasone showed contrary effects and a return to the control state. This may indicate that the M rac extract follows a different mode of action compared to dexamethasone. On the other hand, paracrine activities as determined by the secretion of cytokines/chemokines were attenuated by M rac and dexamethasone in a similar fashion.

## Supporting Information

S1 File Detailed description of the toxicological experiments. (PDF)

S1 Table Test strains used for the Ames test and their characteristics. (PDF)

S2 Table Results of the AMES assays with an extract of *M rac* in the absence (-S9) and the presence (+S9) of metabolic activation. (PDF)

S3 Table Impact of an extract of the mould *Mucor racemosus* on the vitality of peripheral human lymphocytes in absence and presence of metabolic activation and of human derived hepatoma cells (HepG2). (PDF)

S4 Table The protein description, peptide modified sequences and MRM-parameter are shown for interleukin 6, growth-regulated alpha protein and C-X-C motif chemokines 5, 6, and 8 as well as four standard peptides. (PDF)

S5 Table The peak areas of the quantifier transitions are shown for the respective investigated analytes by targeted MRM on an Agilent 6490. The peak areas were calculated by two biological and three technical replicates.
(PDF)

S6 Table The concentration ratios and the p-values associated with these changes are displayed of selected metabolites of the AbsoluteIDQ p180 kit from two biological and two technical replicates. The analysis was performed on an AB Sciex Qtrap 4000 in LC- and FIA-MRM modes. IL-1β = inflammatory activated; dex = inflammatory activated and treated with dexamethasone; M rac = inflammatory activated and treated with the M rac extract. The significantly regulated metabolites by M rac treatment are highlighted bold. lysoPC = lysophosphatidylcholine; PC phosphatidylcholine; SM(OH) = hydroxysphingomyelin, SM; sphingomyelin.
(PDF)

## References

[1] Krutzik PO, Nolan GP (2006) Fluorescent Cell Barcoding in Flow Cytometry Allows High-Throughput Drug Screening and Signaling Profiling. Nat Methods 3: 361–368. 16628206 10.1038/nmeth872

[2] Bauer UE, Briss PA, Goodman RA, Bowman BA (2014) Prevention of Chronic Disease in the 21st Century: Elimination of the Leading Preventable Causes of Premature Death and Disability in the USA. Lancet 384: 45–52. 10.1016/S0140-6736(14)60648-6 24996589

[3] E. BL, Hsu YC, Lee JA (2014) Consideration of the Cellular Microenvironment: Physiologically Relevant Co-Culture Systems in Drug Discovery. Adv Drug Delivery Rev 69–70: 190–204. 10.1016/j.addr.2014.01.013 24524933

[4] Demain AL (1999) Pharmaceutically Active Secondary Metabolites of Microorganisms. Appl Microbiol Biotechnol 52: 455–463. 10570792 10.1007/s002530051546

[5] Arya P, Joseph R, Chou DT (2002) Toward High-Throughput Synthesis of Complex Natural Product-Like Compounds in the Genomics and Proteomics Age. Chem Biol 9: 145–156. 11880029 10.1016/s1074-5521(02)00105-9

[6] Smeekens SP, Gresnigt MS, Becker KL, Cheng SC, Netea SA, Jacobs L, et al (2015) An Anti-Inflammatory Property of Candida Albicans Beta-Glucan: Induction of High Levels of Interleukin-1 Receptor Antagonist Via a Dectin-1/Cr3 Independent Mechanism. Cytokine 71: 215–212. 10.1016/j.cyto.2014.10.013 25461401 PMC4437193

[7] Toledo TR, Dejani NN, Monnazzi LG, Kossuga MH, Berlinck RG, Sette LD, et al (2014) Potent Anti-Inflammatory Activity of Pyrenocine a Isolated from the Marine-Derived Fungus Penicillium Paxilli Ma(G)K. Mediators Inflamm 2014: 767061 10.1155/2014/767061 24574582 PMC3916108

[8] Chen G, Yang X, Nong S, Yang M, Xu B, Zhang W (2013) Two Novel Hydroperoxylated Products of 20(S)-Protopanaxadiol Produced by Mucor Racemosus and Their Cytotoxic Activities against Human Prostate Cancer Cells. Biotechnol Lett 35: 439–443. 10.1007/s10529-012-1098-x 23183919

[9] Tajdini F, Amini MA, Nafissi-Varcheh N, Faramarzi MA (2010) Production, Physiochemical and Antimicrobial Properties of Fungal Chitosan from Rhizomucor Miehei and Mucor Racemosus. Int J Biol Macromol 47: 180–183. 10.1016/j.ijbiomac.2010.05.002 20471417

[10] Haudek-Prinz VJ, Klepeisz P, Slany A, Griss J, Meshcheryakova A, Paulitschke V, et al (2012) Proteome Signatures of Inflammatory Activated Primary Human Peripheral Blood Mononuclear Cells. J Proteomics 76 Spec No.: 150–162. 10.1016/j.jprot.2012.07.012 22813876 PMC3509337

[11] Bileck A, Kreutz D, Muqaku B, Slany A, Gerner C (2014) Comprehensive Assessment of Proteins Regulated by Dexamethasone Reveals Novel Effects in Primary Human Peripheral Blood Mononuclear Cells. J Proteome Res 13: 5989–6000. 10.1021/pr5008625 25347463

[12] Muqaku B, Slany A, Bileck A, Kreutz D, Gerner C (2015) Quantification of Cytokines Secreted by Primary Human Cells Using Multiple Reaction Monitoring: Evaluation of Analytical Parameters. Anal Bioanal Chem 407: 6525–6536. 10.1007/s00216-015-8817-9 26092402

[13] Koal T, Deigner HP (2010) Challenges in Mass Spectrometry Based Targeted Metabolomics. Curr Mol Med 10: 216–226. 20196726 10.2174/156652410790963312

[14] Rabilloud T, Strub JM, Luche S, van Dorsselaer A, Lunardi J (2001) A Comparison between Sypro Ruby and Ruthenium Ii Tris (Bathophenanthroline Disulfonate) as Fluorescent Stains for Protein Detection in Gels. Proteomics 1: 699–704. 11678039 10.1002/1615-9861(200104)1:5<699::AID-PROT699>3.0.CO;2-C

[15] Slany A, Haudek-Prinz V, Zwickl H, Stattner S, Grasl-Kraupp B, Gerner C (2013) Myofibroblasts Are Important Contributors to Human Hepatocellular Carcinoma: Evidence for Tumor Promotion by Proteome Profiling. Electrophoresis 34: 3315–3325. 10.1002/elps.201300326 24115093

[16] Slany A, Haudek-Prinz V, Zwickl H, Stattner S, Grasl-Kraupp B, Gerner C (2013) Myofibroblasts Are Important Contributors to Human Hepatocellular Carcinoma: Evidence for Tumor Promotion by Proteome Profiling. Electrophoresis 34: 3315–3325. 10.1002/elps.201300326 24115093

[17] MacLean B, Tomazela DM, Shulman N, Chambers M, Finney GL, Frewen B, et al (2010) Skyline: An Open Source Document Editor for Creating and Analyzing Targeted Proteomics Experiments. Bioinformatics 26: 966–968. 10.1093/bioinformatics/btq054 20147306 PMC2844992

[18] Gerner C, Vejda S, Gelbmann D, Bayer E, Gotzmann J, Schulte-Hermann R, et al (2002) Concomitant Determination of Absolute Values of Cellular Protein Amounts, Synthesis Rates, and Turnover Rates by Quantitative Proteome Profiling. Mol Cell Proteomics 1: 528–537. 12239281 10.1074/mcp.m200026-mcp200

[19] van Hinsbergh VW (2012) Endothelium—Role in Regulation of Coagulation and Inflammation. Semin Immunopathol 34: 93–106. 10.1007/s00281-011-0285-5 21845431 PMC3233666

[20] Ishihama Y, Oda Y, Tabata T, Sato T, Nagasu T, Rappsilber J, et al (2005) Exponentially Modified Protein Abundance Index (Empai) for Estimation of Absolute Protein Amount in Proteomics by the Number of Sequenced Peptides Per Protein. Mol Cell Proteomics 4: 1265–1272. 15958392 10.1074/mcp.M500061-MCP200

[21] Garcia-Faroldi G, Sanchez-Jimenez F, Fajardo I (2009) The Polyamine and Histamine Metabolic Interplay in Cancer and Chronic Inflammation. Curr Opin Clin Nutr Metab Care 12: 59–65. 10.1097/MCO.0b013e328314b9ac 19057189

[22] Aoki T, Narumiya S (2012) Prostaglandins and Chronic Inflammation. Trends Pharmacol Sci 33: 304–311. 10.1016/j.tips.2012.02.004 22464140

[23] Dutta P, Das S (2015) Mammalian Antimicrobial Peptides: Promising Therapeutic Targets against Infection and Chronic Inflammation. Curr Top Med Chem. 26139111 10.2174/1568026615666150703121819

[24] Filer A, Pitzalis C, Buckley CD (2006) Targeting the Stromal Microenvironment in Chronic Inflammation. Curr Opin Pharmacol 6: 393–400. 16682252 10.1016/j.coph.2006.03.007 PMC3119430

[25] Zhu S, Ashok M, Li J, Li W, Yang H, Wang P, et al (2009) Spermine Protects Mice against Lethal Sepsis Partly by Attenuating Surrogate Inflammatory Markers. Mol Med 15: 275–282. 10.2119/molmed.2009.00062 19593412 PMC2707519

[26] Aiyar N, Disa J, Ao Z, Ju H, Nerurkar S, Willette RN, et al (2007) Lysophosphatidylcholine Induces Inflammatory Activation of Human Coronary Artery Smooth Muscle Cells. Mol Cell Biochem 295: 113–120. 16896535 10.1007/s11010-006-9280-x

[27] Kaur N, Sharma N, Gupta AK (1989) Effects of Dexamethasone on Lipid Metabolism in Rat Organs. Indian J Biochem Biophys 26: 371–376. 2632360

[28] Sugimura K, Fukumoto Y, Satoh K, Nochioka K, Miura Y, Aoki T, et al (2012) Percutaneous Transluminal Pulmonary Angioplasty Markedly Improves Pulmonary Hemodynamics and Long-Term Prognosis in Patients with Chronic Thromboembolic Pulmonary Hypertension. Circ J 76: 485–488. 22185711 10.1253/circj.cj-11-1217

