# Supplementary material for: Proteomic and Metabolomic Analyses Reveal Contrasting Anti-Inflammatory Effects of an Extract of Mucor Racemosus Secondary Metabolites Compared to Dexamethasone
Source: PLoS One. 2015 Oct 23;10(10):e0140367. doi: 10.1371/journal.pone.0140367 (PMC4619718; doi:10.1371/journal.pone.0140367)
Supplement: S1 File — (PDF) [file pone.0140367.s001.pdf]

Experimental Supporting Information

Ames Assay. The indicator strains TA 100, TA 1535, TA 1537 were gift of T. Grummt (UBA, Bad Elster, Germany) while the strains TA 98 and TA 102 were obtained from B. Majer (JTI, Vienna, Austria). Deep frozen cultures were prepared as described by Maron and Ames (1984) with DMSO (1.5 ml portions in cryotubes containing 10% DMSO) and were stored deep frozen at –80 °C. New master plates were made fresh every four to six weeks and stored refrigerated at 4 °C in the dark. The characteristics of the strains (rfa, uvrB, pKM101, pAQ1) were tested as described by Ames *et al.* before the experiments [1].

The Ames tests were carried out as plate incorporation assays [2]. Selective media plates were made 48 h before use and stored at room temperature, in the dark. Overnight cultures (ONC) were made in 3 mL of Oxoid broth No. 2 in polyester tubes (15 mL, Sarstedt, Germany) which were incubated in a rotary shaker (IKA® Schüttler MTS4, Germany), at 37 °C overnight (at least 10 h). In the mutagenicity tests, 0.1 mL of the respective ONCs was plated together with 2.5 mL of top agar, different concentrations of the test compound and 0.5 mL PBS (pH 7.4). Plates were incubated in the dark for 48 h before they were evaluated by manual counting. In experiments with metabolic activation, 0.5 mL of PBS was replaced by the respective amount of S9 mix. In all experiments, activation mix (S9) was prepared according to the recipe for the for standard mix [2], stored on ice and used within 2 h. Per experimental point, three plates were made in parallel. In all experiments, positive controls were included which were dissolved in DMSO.

Statistical significance was tested by one-way analysis of variance (Dunett´s test); in addition also the “two-fold rule” was applied to evaluate the results [3].

Viability of Human Peripheral Lymphocytes. The cells were isolated from the blood of a healthy donor (♂, 35 years) by gradient centrifugation with Histopaque®-1077, washed twice and transferred into RPMI medium [4]. Subsequently they were cultivated in 37 °C at 5% CO_2 atmosphere for 3 h.

Lymphocytes (5 x 105) were seed with 750 µL RPMI medium with 10% FBS, 1% L-glutamine, 1% sodium pyruvate in 6.0 mL polystyrene tubes (Becton Dickinson, Heidebrg, Germany) and incubated for 3 h at 37 °C in a CO_2 incubator (Napco Water Jacketed CO_2 incubator, Fisher Scientific Inc., USA; 5% CO_2, 95% humidity) in presence or absence of 1% metabolic activation mix (S9) and with the tests solution [2]. After treatment, 650 µL of medium were carefully removed. The medium with cells were directly used for the determination of the viability. Finally, 20 µL of the cell suspensions were mixed with 20 µL trypan blue solution (4%). 20 µL of this mix were pipetted into a Neubauer chamber (Tiefe Deph CE, Profoudeur 0,0025 mm^2, Labor Optik, Germany) and the numbers of blue and colorless cell determined under a light microscope (100×magnification, Nikon TMS Inverted Microscope, Japan). MMS (50.0 mg/mL, -S9) was used as a positive control in experiments without metabolic activation and in experiments with metabolic activation AFB1 (100.0 µg/mL).

For each experimental point three cultures were tested in parallel, and from each at least 300 cells were evaluated. The result of the experiments were analyzed with one-way ANOVA followed by Dunnett's multiple comparison test ($p<0.05$).

Viability of Human Peripheral Lymphocytes. The cells were kindly provided by F. Darroudi (Univ. Leiden, The Netherlands). The cells were stored in aliquots of 2 mL portions in liquid nitrogen. Subclones were made freshly from the deep frozen cultures for the test.

These subcultures were used to 3-4 passages. The cultivation of the cells has been described in detail by Ehrlich *et al.* [5].

The cells (4 x 105 per well) were seeded into 12 well plates (BD Falcon™ 12-well Cell Culture Plate) and incubated at 37 °C in CO_2 incubator (Napco Water Jacketed CO_2 incubator, Fisher Scientific Inc., USA; 5% CO_2, 95% humidity) with DMEM supplemented with 15% FCS and 1% penicillin/streptomycin. After 24 h, different amounts of the extract were added. Subsequently, the cells were cultivated for 24 h then the medium was removed and collected. The cells was trypsinised (500 μL of trypsin solution per well). The cell suspensions as well as the supernatant were combined and collected in 15 mL polypropylene tubes (Sarstedt, Germany) and centrifuged (800 rpm for 4 min). Subsequently, the supernatants were discarded, and the pellets were resuspended in 2.5 mL of PBS (Dulbeccos phosphate buffer, pH 7.4, Sigma Aldrich, Germany). The cells were separated by pressing the suspensions through a syringe (needle: Microlance3® 0.4 × 19, Becton Dickinson, Basel, Switzerland). Finally, 20 μL of the cell suspension was mixed with 20 μL trypan blue solution. An aliquot of 20 μL of this mixture was pipetted into a Neubauer chamber (Tiefe Deph CE, Profoudeur 0,0025 mm^2, Labor Optik, Germany) and the numbers of blue and colorless cells was determined under a light microscope (100× magnification, Nikon TMS Inverted Microscope, Japan).

The result of the experiments were analyzed with one-way ANOVA followed by Dunnett's multiple comparison test ($p<0.05$).

References

1. Ames BN, Lee FD, Durston WE (1973) An Improved Bacterial Test System for the Detection and Classification of Mutagens and Carcinogens. Proc Natl Acad Sci 70: 782-786.
2. Maron DM, Ames BN (1984) Revised Methods for the Salmonella Mutagenicity Test; Kilbey BJ, Legator M, Nocols W, Ramel C, editors. New York: Elsevier Science Publishers BV.
3. Kirkland DJ (1990) Basic Mutagenicity Tests: Ukems Recommended Procedures. Cambridge, New York, Melbourne, Sydney: Cambridge University Press.
4. Fenech M (2007) Cytokinesis-Block Micronucleus Cytome Assay. Nat Protoc 2: 1084-1104.
5. Ehrlich V, Darroudi F, Uhl M, Steinkellner H, Zsivkovits M, et al. (2002) Fumonisin B(1) Is Genotoxic in Human Derived Hepatoma (Hepg2) Cells. Mutagenesis 17: 257-260.
