# Supplementary material for: Proteomic and Metabolomic Analyses Reveal Contrasting Anti-Inflammatory Effects of an Extract of Mucor Racemosus Secondary Metabolites Compared to Dexamethasone
Source: PLoS One. 2015 Oct 23;10(10):e0140367. doi: 10.1371/journal.pone.0140367 (PMC4619718; doi:10.1371/journal.pone.0140367)
Supplement: S1 Table — (PDF) [file pone.0140367.s002.pdf]

S. typh. strain	Gene affected[1]	DNA repair[2]	LPS[3]	Biotin requirement[4]	Plasmids[5]	Mutational Event	Range of his$^+$ colonies[6]
**TA98**	*hisD3052*	*uvrB*	*rfa*	*bio-*	pKM101	frameshift	15–60
**TA100**	*hisG46*	*uvrB*	*rfa*	*bio-*	pKM101	base-pair substitution	75–200
**TA102**	*hisG428*	-	*rfa*	*bio-*	pKM101, pAQ1	base-pair substitution	240–360
**TA1535**	*hisG46*	*uvrB*	*rfa*	*bio-*	-	base-pair substitution	3–37
**TA1537**	*hisC3076*	*uvrB*	*rfa*	*bio-*	-	frameshift	4–31

[1] Target genes in the different strains, leading to histidine requirement.

[2] *uvrB* - Deleted excision repair system, increasing the sensitivity.

[3] The *rfa* mutation changes the properties of the bacterial cell wall and results in partial loss of the lipopolysaccharide (LPS) barrier and increases the permeability of cells to certain types of chemicals. The *rfa* mutation is indicated by sensitivity to crystal violet.

[4] Requirement for biotin.

[5] pKM101, pAQ1– plasmids enhance sensitivity to special types of mutagens.

[6] Number of background colonies according Ref. [1].
