# Supplementary material for: Proteomic and Metabolomic Analyses Reveal Contrasting Anti-Inflammatory Effects of an Extract of Mucor Racemosus Secondary Metabolites Compared to Dexamethasone
Source: PLoS One. 2015 Oct 23;10(10):e0140367. doi: 10.1371/journal.pone.0140367 (PMC4619718; doi:10.1371/journal.pone.0140367)
Supplement: S2 Table — (PDF) [file pone.0140367.s003.pdf]

AMES assays of an extract of Mucor racemosus in the absence of metabolic activation (-S9)[1]										
**Dose (µL/plate)**	**TA 98 (mean±S.D.)**		**TA 100 (mean±S.D.)**		**TA 102 (mean±S.D.)**		**TA 1535 (mean±S.D.)**		**TA 1537 (mean±S.D.)**	
	**Exp 1**	**Exp 2**	**Exp 1**	**Exp 2**	**Exp 1**	**Exp 2**	**Exp 1**	**Exp 2**	**Exp 1**	**Exp 2**
**0**	26±9	30±4.7	87±12	87±12	260±23	297±33	11±6	11±6	9±3	9±5
**50**	27±2	26±8	89±10	93±21	308±20	307±13	12±5	9±2	10±2	10±2
**100**	24±6	28±10	97±12	94±14	311±13	309±33	14±3	11±4	12±3	10±4
**175**	26±11	29±2	115±6	76±6	277±45	299±30	12±2	11±4	10±3	9±2
**500**	23±2	26±6	108±24	92±13	260±30	329±10	15±2	13±4	13±1	11±4
**Positive control[2]**	825±170*	763±136*	567±84*	557±35*	3333±496*	3467±465*	533±85*	620±30*	48±7*	53±11*
**AMES assays of an extract of Mucor racemosus in the presence of metabolic activation (+S9)[1]**										
**Dose (µL/plate)**	**TA 98 (mean±S.D.)**		**TA 100 (mean±S.D.)**		**TA 102 (mean±S.D.)**		**TA 1535 (mean±S.D.)**		**TA 1537 (mean±S.D.)**	
	**Exp 1**	**Exp 2**	**Exp 1**	**Exp 2**	**Exp 1**	**Exp 2**	**Exp 1**	**Exp 2**	**Exp 1**	**Exp 2**
**0**	22±9	26±2	103±14	109±10	275±9	295±42	9±1	5±2	10±3	10±4
**50**	23±4	28±2	100±11	94±13	315±12	303±30	7±1	7±2	11±2	10±2
**100**	24±4	25±4	93±8	99±11	306±23	307±13	7±2	7±3	11±4	10±4
**175**	19±5	25±7	111±4	95±13	272±34	301±14	7±1	8±4	9±5	9±2
**500**	32.9	27±4	98±16	75±14	334±70	341±38	7±1	7±4	11±3	11±4
**Positive control[2]**	419.2*	428±19*	727±106*	698±85*	1875±483*	1880±380*	249±28*	277±42*	272±10*	290±18*

[1] The tested extract obtained from Syntrion Co. was diluted 1:10 with PBS (pH 7.4) to obtain the stock solution used in the experiments.
[2] The positive controls were TNF (2,4,7-trinitro-9-fluorenone, 0.1 µg/plate) for TA98; NaN3 (sodium azide, 1.5 µg/plate) for TA100; MMS (methylmethanesulfonate, 1.0 µl/plate) for TA102; NaN3 (sodium azide, 1.5 µg/plate) for TA1535; ICR-191 (5.0 µg/plate) for TA1537.
*Statistical significance (p<0.05, Dunett´s test).
