# Supplementary material for: Proteomic and Metabolomic Analyses Reveal Contrasting Anti-Inflammatory Effects of an Extract of Mucor Racemosus Secondary Metabolites Compared to Dexamethasone
Source: PLoS One. 2015 Oct 23;10(10):e0140367. doi: 10.1371/journal.pone.0140367 (PMC4619718; doi:10.1371/journal.pone.0140367)
Supplement: S3 Table — (PDF) [file pone.0140367.s004.pdf]

	Peripheral human lymphocytes		HepG2
**Concentration (µL/mL)**	**Total viability –S9[1] (%, Mean ±S.D.)**	**Total viability +S9[2] (%, Mean ±S.D.)**	**Total viability[3] (%, Mean ±S.D.)**
**0.0**	99.6±0.1	98.7±0.8	98.0±0.7
**0.1**	99.5±0.1	99.0±0.1	98.3±0.3
**1.1**	99.2±0.1	99.1±0.2	98.8±0.3
**3.3**	99.3±0.5	99.0±0.2	96.9±0.6
**10**	98.4±0.5	98.5±0.6	98.1±0.8
**pos. control**	MMS[4] = 41.4±5.2*	AFB_1[4] = 71.7±1.3*	MMS[5] = 66.9±4.9*

[1]The cells treated with *Mucor racemossus* extract for 3 h. Subsequently, the vitality was evaluated with the trypan blue exclusion technique.

[2]The cells treated with *Mucor racemossus* extract for 3 h in presence of metabolic activation mix (1.0%, +S9). Subsequently, the vitality was evaluated with the trypan blue exclusion technique.

[3]The cells treated with *Mucor racemossus* extract for 24 h. Subsequently, the vitality was evaluated with the trypan blue exclusion technique.

[4]MMS (50.0 mg/mL) and AFB_1 (100.0 mg/mL) was used as positive control (3 h exposure).

[5]MMS (200.0 mg/mL) was used as positive control (2h exposure).

*$p<0.05$ (Dunnett’s multiple comparison test)
