# Supplementary material for: Proteomic and Metabolomic Analyses Reveal Contrasting Anti-Inflammatory Effects of an Extract of Mucor Racemosus Secondary Metabolites Compared to Dexamethasone
Source: PLoS One. 2015 Oct 23;10(10):e0140367. doi: 10.1371/journal.pone.0140367 (PMC4619718; doi:10.1371/journal.pone.0140367)
Supplement: S4 Table — (PDF) [file pone.0140367.s005.pdf]

Accession	Gene	Protein Name	Peptide Modified Sequence	Precursor [m/z]	z (+)	Coll. En. (eV)	Product [m/z]	z (+)	Fragment Ion
P05231	IL6	Interleukin-6	EALAENNLNLPK	663.3566	2	19.1	1012.5422	1	y9
P05231	IL6	Interleukin-6	EALAENNLNLPK	663.3566	2	19.1	941.5051	1	y8
P05231	IL6	Interleukin-6	EALAENNLNLPK	663.3566	2	19.1	812.4625	1	y7
P09341	CXCL1	Growth-regulated alpha Protein	AC[+57]LNPASPIVK	585.3210	2	16.3	938.5669	1	y9
P09341	CXCL1	Growth-regulated alpha Protein	AC[+57]LNPASPIVK	585.3210	2	16.3	825.4829	1	y8
P09341	CXCL1	Growth-regulated alpha Protein	AC[+57]LNPASPIVK	585.3210	2	16.3	711.4400	1	y7
P10145	CXCL8	Interleukin-8	VIESGPHC[+57]ANTEIIVK	589.6417	3	16.4	834.4247	2	y15
P10145	CXCL8	Interleukin-8	VIESGPHC[+57]ANTEIIVK	589.6417	3	16.4	777.8827	2	y14
P10145	CXCL8	Interleukin-8	VIESGPHC[+57]ANTEIIVK	589.6417	3	16.4	713.3614	2	y13
P42830	CXCL5	C-X-C motif chemokine 5	C[+57]VC[+57]LQTTQGVHPK	509.9184	3	13.6	766.4206	1	y7
P42830	CXCL5	C-X-C motif chemokine 5	C[+57]VC[+57]LQTTQGVHPK	509.9184	3	13.6	537.3144	1	y5
P42830	CXCL5	C-X-C motif chemokine 5	C[+57]VC[+57]LQTTQGVHPK	509.9184	3	13.6	634.8244	2	y11
P80162	CXCL6	C-X-C motif chemokine 6	QVC[+57]LDPEAPFLK	708.8632	2	20.7	1189.5922	1	y10
P80162	CXCL6	C-X-C motif chemokine 6	QVC[+57]LDPEAPFLK	708.8632	2	20.7	916.4775	1	y8
P80162	CXCL6	C-X-C motif chemokine 6	QVC[+57]LDPEAPFLK	708.8632	2	20.7	801.4505	1	y7
P80162	CXCL6	C-X-C motif chemokine 6	QVC[+57]LDPEAPFLK	708.8632	2	20.7	504.3180	1	y4
