# Supplementary material for: Proteomic and Metabolomic Analyses Reveal Contrasting Anti-Inflammatory Effects of an Extract of Mucor Racemosus Secondary Metabolites Compared to Dexamethasone
Source: PLoS One. 2015 Oct 23;10(10):e0140367. doi: 10.1371/journal.pone.0140367 (PMC4619718; doi:10.1371/journal.pone.0140367)
Supplement: S5 Table — The peak areas were calculated by two biological and three technical replicates. (PDF) [file pone.0140367.s006.pdf]

	control		IL-1b		IL-1b + dexamethasone		IL-1b + M rac	
	**Exp. 1**	**Exp. 2**	**Exp. 1**	**Exp. 2**	**Exp. 1**	**Exp. 2**	**Exp. 1**	**Exp. 2**
**IL6**	9±12	9±6	4298±121	1016±148	2417±242	290±53	3222±294	569±30
**GROA**	59±46	23±12	2532±171	1728±214	449±76	837±125	2330±119	465±96
**CXCL5**	2896±547	1910±44	3847±556	7157±158	312±160	2627±38	2504±429	1388±32
**CXCL6**	22±25	6±2	2595±137	545±51	529±44	211±25	1937±126	144±25
**CXCL8**	2316±258	648±103	108504±12659	37111±1573	7379±1071	9141±540	67819±7795	9285±137
**HK0**[a]	97578±6076	86376±565	92170±6473	86977±4198	95610±6679	105876±5094	86324±7681	88242±4305
**HXK1**[a]	11583±439	10097±422	11077±185	10491±599	10750±941	12469±1161	10208±794	9973±761
**Glu1-Frib**[a]	6962±269	5670±61	6593±635	6028±256	6593±82	6716±122	6219±495	5475±112

[a] Peptide standard
