# Supplementary material for: Proteomic and Metabolomic Analyses Reveal Contrasting Anti-Inflammatory Effects of an Extract of Mucor Racemosus Secondary Metabolites Compared to Dexamethasone
Source: PLoS One. 2015 Oct 23;10(10):e0140367. doi: 10.1371/journal.pone.0140367 (PMC4619718; doi:10.1371/journal.pone.0140367)
Supplement: S6 Table — The analysis was performed on an AB Sciex Qtrap 4000 in LC- and FIA-MRM modes. IL-1β = inflammatory activated; dex = inflammatory activated and treated with dexamethasone; M rac = inflammatory activated and treated with the M rac extract. The significantly regulated metabolites by M rac treatment are highlighted bold. lysoPC = lysophosphatidylcholine; PC phosphatidylcholine; SM(OH) = hydroxysphingomyelin, SM; sphingomyelin. (PDF) [file pone.0140367.s007.pdf]

Analyte	concentration ratios[a] IL-1b/con	dex/IL-1b	M rac/IL-1b	p-values[b] IL-1b/con	dex/IL-1b	M rac/IL-1b
acetyl-L-carnitine	0.70	-0.97	0.99	3.44E-04	8.50E-01	9.18E-01
**arginine**	0.90	-0.89	0.87	8.39E-02	1.83E-01	2.90E-02
histidine	0.90	-0.77	0.85	3.00E-01	7.62E-03	8.82E-02
**spermine**	0.88	-0.86	0.70	1.34E-01	9.04E-02	1.33E-02
taurine	0.12	-1.32	0.85	7.38E-05	1.07E-01	3.12E-01
sum of hexoses	0.73	-0.67	0.94	6.78E-02	1.24E-03	3.19E-01
lysoPC acyl C14:0	0.71	-0.79	1.34	1.57E-01	1.82E-01	2.32E-01
**lysoPC acyl C16:0**	0.99	-1.59	1.85	9.72E-01	3.06E-02	8.19E-03
lysoPC acyl C16:1	0.71	-0.87	1.64	9.47E-02	4.69E-01	1.56E-01
**lysoPC acyl C17:0**	1.10	-1.34	1.35	5.73E-01	5.26E-02	1.27E-01
**lysoPC acyl C18:0**	1.89	-1.87	2.07	5.30E-02	1.11E-01	2.29E-03
lysoPC acyl C18:1	0.72	-1.20	1.87	2.13E-01	2.55E-01	2.69E-02
lysoPC acyl C18:2	0.75	-0.69	1.79	1.06E-01	4.45E-01	7.24E-02
lysoPC acyl C20:3	0.72	-1.12	1.77	1.40E-01	5.52E-01	5.32E-02
lysoPC acyl C20:4	0.68	-1.07	1.69	3.87E-02	7.81E-01	8.23E-02
lysoPC acyl C24:0	1.59	-1.44	1.50	1.24E-01	1.84E-01	2.00E-01
lysoPC acyl C26:0	1.60	-1.90	1.66	1.80E-01	1.11E-01	9.92E-02
lysoPC acyl C26:1	1.53	-1.51	1.48	1.20E-01	1.30E-01	1.15E-01
lysoPC acyl C28:0	2.05	-3.17	1.60	1.32E-01	7.00E-02	1.02E-01
lysoPC acyl C28:1	1.67	-1.72	1.53	1.62E-01	1.47E-01	1.23E-01
PC diacyl C24:0	0.90	-0.44	1.46	6.18E-01	9.38E-02	1.91E-01
PC diacyl C26:0	1.05	-0.60	1.47	7.49E-01	1.27E-01	2.90E-01
PC diacyl C28:1	1.00	-0.72	1.31	9.81E-01	1.72E-01	1.88E-01
**PC diacyl C30:0**	1.77	-2.94	1.33	1.16E-02	2.63E-03	3.22E-02
PC diacyl C30:2	1.33	-1.59	1.23	1.70E-01	1.83E-01	1.86E-01
PC diacyl C32:0	2.46	-4.76	1.49	2.52E-02	9.75E-03	9.85E-02
PC diacyl C32:1	2.11	-2.87	1.34	6.59E-02	3.48E-02	2.02E-01
PC diacyl C32:2	1.97	-2.88	1.30	9.02E-02	4.11E-02	2.91E-01
PC diacyl C32:3	2.03	-2.81	1.25	9.48E-02	4.85E-02	4.06E-01
PC diacyl C34:1	2.10	-3.96	1.34	4.55E-02	1.45E-02	1.40E-01
PC diacyl C34:2	1.94	-2.77	1.28	6.13E-02	2.59E-02	2.42E-01
PC diacyl C34:3	2.04	-2.89	1.25	6.89E-02	3.36E-02	3.40E-01
PC diacyl C34:4	2.14	-2.81	1.22	9.33E-02	5.64E-02	4.55E-01
PC diacyl C36:0	1.28	-2.10	1.14	4.10E-01	5.70E-02	5.26E-01
**PC diacyl C36:1**	2.04	-5.04	1.51	2.03E-02	4.30E-03	1.98E-02
PC diacyl C36:2	1.75	-3.44	1.28	5.43E-02	9.69E-03	1.46E-01
PC diacyl C36:3	2.05	-3.12	1.27	4.74E-02	1.85E-02	2.38E-01
PC diacyl C36:4	2.09	-2.61	1.25	5.22E-02	3.90E-02	2.86E-01
PC diacyl C36:5	2.07	-2.91	1.21	7.97E-02	4.07E-02	4.36E-01
PC diacyl C36:6	2.07	-2.84	1.02	1.03E-01	5.62E-02	9.50E-01
PC diacyl C38:0	1.47	-2.84	1.16	1.85E-01	1.68E-02	4.30E-01
PC diacyl C38:1	1.41	-2.77	1.71	1.67E-01	2.78E-03	6.91E-02
PC diacyl C38:3	1.71	-3.75	1.32	5.93E-02	7.79E-03	1.02E-01
PC diacyl C38:4	1.85	-3.34	1.28	4.29E-02	9.13E-03	1.51E-01
PC diacyl C38:5	1.97	-3.49	1.23	4.99E-02	1.60E-02	3.00E-01
PC diacyl C38:6	2.22	-3.22	1.28	4.63E-02	2.19E-02	2.53E-01
**PC diacyl C40:1**	1.22	-1.33	1.53	8.40E-02	3.57E-02	3.04E-02
**PC diacyl C40:2**	1.37	-2.60	1.72	1.62E-01	1.10E-02	6.99E-03

**PC diacyl C40:3**	1.55	-4.40	1.47	1.14E-01	1.26E-02	4.21E-02
PC diacyl C40:4	1.77	-4.48	1.29	5.68E-02	6.98E-03	1.17E-01
PC diacyl C40:5	1.81	-4.13	1.34	5.19E-02	1.05E-02	1.05E-01
PC diacyl C40:6	2.21	-3.85	1.23	2.92E-02	1.14E-02	2.42E-01
PC diacyl C42:0	1.35	-1.68	1.80	1.43E-01	6.58E-02	2.26E-01
PC diacyl C42:1	1.54	-2.32	1.87	1.03E-01	1.81E-02	1.85E-01
**PC diacyl C42:2**	1.23	-2.23	1.85	2.53E-01	9.69E-03	2.81E-02
PC diacyl C42:4	1.98	-4.19	1.27	2.88E-02	8.93E-03	1.80E-01
PC diacyl C42:5	1.56	-3.39	1.36	5.26E-02	6.05E-03	5.01E-02
PC diacyl C42:6	1.54	-2.27	1.32	8.47E-02	1.36E-02	6.92E-02
**PC acyl alkyl C30:0**	1.54	-1.70	1.24	5.29E-03	2.73E-03	2.42E-02
PC acyl alkyl C30:1	1.38	-1.35	1.14	1.89E-02	1.01E-02	1.21E-01
PC acyl alkyl C30:2	1.24	-0.93	1.09	1.88E-02	5.28E-01	4.71E-01
PC acyl alkyl C32:1	2.04	-2.91	1.40	6.68E-02	3.12E-02	1.37E-01
PC acyl alkyl C32:2	1.71	-2.26	1.31	1.30E-01	6.30E-02	2.46E-01
PC acyl alkyl C34:0	2.12	-3.59	1.46	3.26E-02	1.15E-02	9.14E-02
PC acyl alkyl C34:1	2.02	-3.63	1.35	5.33E-02	1.75E-02	1.39E-01
PC acyl alkyl C34:2	1.88	-2.85	1.34	6.79E-02	2.43E-02	1.73E-01
PC acyl alkyl C34:3	1.72	-2.08	1.29	1.43E-01	9.00E-02	3.09E-01
PC acyl alkyl C36:0	1.78	-2.70	1.00	1.55E-01	5.49E-02	9.86E-01
PC acyl alkyl C36:1	2.03	-3.94	1.39	3.77E-02	1.19E-02	8.17E-02
PC acyl alkyl C36:2	1.95	-3.29	1.24	5.01E-02	1.62E-02	2.40E-01
PC acyl alkyl C36:3	1.79	-3.16	1.30	8.41E-02	2.31E-02	2.00E-01
PC acyl alkyl C36:4	1.30	-2.13	1.37	2.87E-01	5.31E-02	1.27E-01
PC acyl alkyl C36:5	1.40	-2.27	1.35	1.95E-01	4.02E-02	1.52E-01
PC acyl alkyl C38:0	1.92	-3.07	1.20	8.30E-02	3.27E-02	4.90E-01
PC acyl alkyl C38:1	1.73	-3.55	1.28	6.22E-02	9.12E-03	1.76E-01
PC acyl alkyl C38:2	1.88	-3.75	1.32	4.14E-02	9.68E-03	1.10E-01
PC acyl alkyl C38:3	1.91	-3.55	1.33	4.57E-02	1.14E-02	1.27E-01
PC acyl alkyl C38:4	1.63	-3.10	1.26	8.19E-02	1.00E-02	1.70E-01
PC acyl alkyl C38:5	1.47	-2.82	1.31	1.60E-01	1.99E-02	2.00E-01
PC acyl alkyl C38:6	1.40	-2.71	1.32	1.93E-01	1.72E-02	2.04E-01
**PC acyl alkyl C40:1**	2.06	-3.16	1.55	4.71E-02	2.01E-02	8.15E-02
PC acyl alkyl C40:2	1.98	-3.38	1.49	1.44E-02	1.25E-03	4.73E-02
PC acyl alkyl C40:3	1.88	-3.44	1.24	4.57E-02	1.30E-02	2.27E-01
PC acyl alkyl C40:4	1.70	-3.35	1.27	9.35E-02	1.80E-02	2.02E-01
PC acyl alkyl C40:5	1.83	-3.48	1.35	3.40E-02	6.34E-03	8.38E-02
PC acyl alkyl C40:6	1.84	-3.48	1.30	5.24E-02	1.28E-02	1.80E-01
PC acyl alkyl C42:0	1.49	-1.84	1.14	7.02E-02	2.17E-02	4.30E-01
PC acyl alkyl C42:1	1.82	-2.86	1.35	5.80E-02	2.00E-02	2.40E-01
PC acyl alkyl C42:2	2.30	-3.32	1.22	4.29E-02	2.26E-02	4.04E-01
PC acyl alkyl C42:3	2.24	-3.49	1.30	2.76E-02	1.16E-02	2.70E-01
PC acyl alkyl C42:4	2.31	-4.22	1.49	2.77E-02	1.18E-02	8.44E-02
PC acyl alkyl C42:5	1.56	-2.04	1.22	3.32E-02	9.70E-03	1.20E-01
PC acyl alkyl C44:3	1.92	-3.26	1.35	3.25E-02	7.52E-03	1.91E-01
PC acyl alkyl C44:4	2.61	-3.53	1.29	3.75E-02	2.77E-02	3.65E-01
PC acyl alkyl C44:5	1.69	-1.56	1.29	1.10E-01	1.55E-01	2.77E-01
PC acyl alkyl C44:6	1.25	-1.68	1.24	5.32E-01	5.68E-02	1.84E-01
SM (OH) C14:1	1.88	-4.65	1.47	7.17E-02	1.84E-02	7.05E-02
SM (OH) C16:1	1.91	-5.12	1.42	1.18E-01	3.36E-02	1.55E-01

**SM (OH) C22:1**	1.33	-1.79	2.98	5.09E-01	2.74E-01	1.48E-02
SM (OH) C22:2	1.75	-7.10	1.47	2.34E-01	5.06E-02	2.87E-01
SM (OH) C24:1	1.53	-5.33	1.57	2.93E-01	2.79E-02	2.04E-01
SM C16:0	1.78	-6.57	1.47	1.34E-01	2.68E-02	1.16E-01
SM C16:1	1.50	-2.20	1.46	1.47E-01	4.22E-02	7.80E-02
SM C18:0	2.06	-5.63	1.44	1.24E-01	3.38E-02	2.21E-01
SM C18:1	1.54	-3.13	1.40	2.62E-01	6.39E-02	3.10E-01
SM C20:2	0.57	-1.11	0.82	5.07E-01	8.88E-01	8.18E-01
SM C24:0	1.57	-6.52	1.83	3.16E-01	4.86E-02	1.32E-01
SM C24:1	1.45	-8.59	1.81	4.10E-01	4.96E-02	1.46E-01
SM C26:0	1.40	-2.98	1.36	5.59E-01	7.38E-02	6.35E-01
**SM C26:1**	0.64	-5.24	3.57	3.61E-01	2.97E-02	2.95E-02

[a] boxes highlighted in red represent a regulation of <0.5, while green boxes represent a down-regulation greater than 2 (dex/IL-1b) or an up-regulation greater than 1.5 (M rac/IL-1b).

[b] boxes highlighted in green represent a p-value <0.05.
